# Implications of Heat Treatment and Systemic Delivery of Foliar-Applied Oxytetracycline on Citrus Physiological Management and Therapy Delivery

**DOI:** 10.3389/fpls.2019.00041

**Published:** 2019-01-30

**Authors:** Christopher Vincent, Myrtho Pierre, Jinyun Li, Nian Wang

**Affiliations:** ^1^Citrus Research and Education Center, Horticultural Sciences Department, Institute of Food and Agricultural Sciences, University of Florida, Lake Alfred, FL, United States; ^2^Citrus Research and Education Center, Department of Microbiology and Cell Science, Institute of Food and Agricultural Sciences, University of Florida, Lake Alfred, FL, United States

**Keywords:** citrus, huanglongbing (HLB), anti-microbial delivery, heat treatment, xenobiotic delivery

## Abstract

Huanglongbing is an economically devastating disease of citrus in Florida and around the world. This study was undertaken to assess two grower-used therapies, heat treatment, and foliar anti-bacterial application. Specifically, there was an industry claim that heat treatment improved subsequent systemic uptake of foliar-applied anti-bacterial compounds. We hypothesized that new vegetative growth induced by heat treatment could lead to increased foliar delivery because of a greater number of new leaves in which cuticles would be more permeable. The study included two factors (1) heat treatment (with or without) and (2) pruning, in which all new leaves, all mature leaves, or no leaves were removed. A commercial formulation of oxytetracycline (OTC) was applied to plants with a non-ionic penetrant surfactant, but one branch on each tree was covered to assess direct versus systemic delivery. The study was repeated twice, destructively assessing whole-plant leaf area and dry weights, as well as OTC content in directly applied and covered leaves. Heat treatment and defoliation treatments reduced growth, but did not affect systemic delivery of OTC. OTC was detected in nearly all covered leaf samples in both repetitions, though at lower concentrations than in directly applied leaves. We conclude that neither heat treatment nor leaf age strongly affect systemic OTC delivery. Implications of this study for leaf age effects on foliar delivery and for phloem delivery of foreign compounds through foliar application are discussed.

## Introduction

Huanglongbing disease (“citrus greening”; HLB) is one of the most economically damaging diseases of perennial crop plants. It is caused by the persistent infection of a phloem-limited bacterium *Candidatus* Liberibacter asiaticus (Las), which leads to hyperaccumulation of carbohydrates in leaves, and starvation of sink tissues (Cimò et al., 2013), resulting in reduced overall growth, leaf senescence, loss of root length and mass density, reduced fruit production and increased pre-harvest fruit drop (Achor et al., 2010; Gottwald et al., 2014; Johnson et al., 2014; Wang et al., 2017). The disease has no cure, and no resistant citrus germplasm has been found. By 2014, 9 years after its detection in Florida, United States, HLB was estimated to have reduced the statewide sweet orange crop by 100 million boxes per year, approximately 4 million metric tons (Spreen et al., 2014).

Because of the economic impacts of HLB, therapies to reduce populations of Las within infected plants in the field have been an area of research as well as grower practice. These therapies include steam or hot-water generated heat treatments and the use of anti-bacterial compounds (Doud et al., 2014; Hu et al., 2017).

Heat treatment, sometimes called “thermotherapy,” has been effective in eliminating Las from citrus without killing the citrus, when applied under controlled conditions (Hoffman et al., 2012). In field studies, Las titer has been reduced but not eliminated with 48 h treatments of 42°C or 1 min treatments of 60°C without killing the plant (Doud et al., 2014; Jia et al., 2017), though assessments of treatments on other plant variables such as photosynthesis were not considered in these studies. In response to these positive results in terms of reducing bacterial titer, some companies have begun to market heat-treatment services in commercial citrus groves.

Although a range of compounds have been assessed for efficacy against Las (Yang et al., 2016), currently there are two active ingredients that are approved for use in citrus under an emergency exemption (EPA, Section 18c) to address the HLB crisis affecting Florida citrus. These are streptomycin and oxytetracycline hydroxide (OTC). Large scale in-field trials of streptomycin and OTC treatments are ongoing (B. Shatters, USDA-ARS, *personal communication*; S. Slinski, Citrus Research and Development Foundation, *personal communication*). Several studies have indicated that OTC and, to a lesser degree, streptomycin can dramatically reduce Las titer when injected into the trunk (Hu and Wang, 2016; Hu et al., 2017), although this application method is not approved for agricultural use. Because these products are new to use in citrus, the degree to which they can be delivered into leaves and the degree to which they become systemic in the plant have not been shown.

The current study responded to claims that heat treatment increased the uptake of foliar-applied anti-bacterial compounds (S. Slinski, Citrus Research and Development Foundation, *personal communication*). Because we observed heat treatments to induce a new flush of vegetative growth, we hypothesized that the new flush could improve anti-microbial uptake. This was based on the observation that young leaves have thinner cuticles and that there are differences in cross-cuticular agrochemical delivery among leaf ages (Orbović et al., 2001). This study was designed to test the effect of treatments on citrus plants, not to test the efficacy of treatments against Las. Although previous work had addressed the movement of OTC after trunk injection, no study had assessed systemic delivery of commercial anti-bacterial compounds after foliar application, which is the only on-label method of application (Hu and Wang, 2016; Hu et al., 2017). Thus we designed and implemented a study to test: (1) the effect of heat treatment on citrus growth and systemic OTC delivery, (2) whether leaf age mediated the heat treatment effect, and (3) the degree of systemic OTC delivery from foliar application.

## Materials and Methods

The same study and treatment design were repeated twice: July 6, 2017–November 17, 2017 (Trial 1) and September 20, 2017–January 26, 2018 (Trial 2).

### Plant Materials and Experimental Setup

Thirty-six ‘Valencia’/X-639 trees at 1 year past budding from a local nursery were repotted in 30-gallon (113 L) plastic containers in sandy soil taken from the field. Three days later, trees were placed in the ground with the top of the container at ground level in the field, in Lake Alfred, FL, United States (28.1021° N, 81.7121° W). The pots allowed us to isolate the entire root system for destructive sampling at the end of the study. Submerging the pots in the soil allowed us to imitate typical field conditions, preventing heating the rootzone any more than would occur in a field setting. Thus we reproduced a typical field heat treatment, while maintaining the ability to assess the entire root system of each tree. Each plant was irrigated 1 h daily with a 4 L h^-1^ drip emitter. A commercial slow-release fertilizer (14-3-11 N-P-K) was applied to the soil surface around each tree at a rate of 17 g of total nitrogen per tree.

### Experimental Design

The experiment was set out as a randomized complete block design with six blocks. The treatment structure was a factorial design with heat treatment and pruning factors. Heat treatment had two levels: (1) with heat treatment (Heat) and (2) without heat treatment (No Heat). Pruning had three levels: (1) old leaves removed with only young leaves remaining (Young Leaves), (2) young leaves removed with only mature leaves remaining (Mature Leaves), and (3) no leaves removed with all leaves remaining (All Leaves). Blocks were arranged in a row of pots. Plants were assigned randomly to block. The experimental unit was an individual plant in a single pot.

### Treatments

**FIGURE 1:**
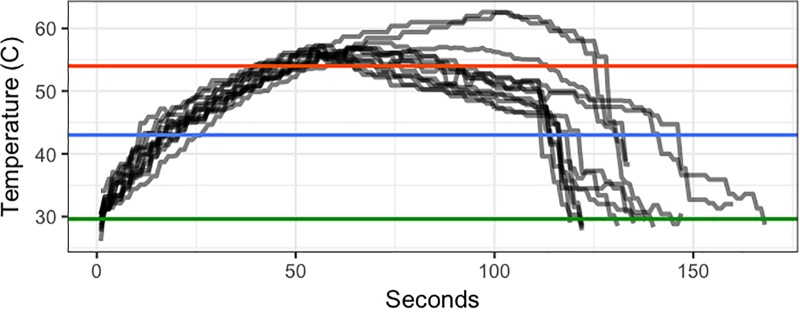
Temperature (°C) of heat treatments over time (seconds after start of heating) in Trial 2. Green line shows the ambient temperature. Blue line shows the minimum threshold target temperature (43°C). Red line shows the maximum target temperature (54°C).

Twenty-five days after repotting and placing trees in the ground, 18 trees were treated with steam-generated heat. The first repetition heat treatment was imposed by a local heat treatment company (Premier Energy, Inc., Woodstock, GA, United States). The parameters of the first repetition heat treatment were heat between 43 and 54°C with the high temperature held for no longer than 45 s. The second repetition heat treatment was imposed by University of Florida heat treatment personnel. Actual temperatures for the second repetition are shown in Figure 1. Generally these temperatures were higher for longer than those of the first repetition. In both repetitions, heat treatment consisted of a tent placed on each treated tree for 60–180 s. Immediately after heat treatment, each tree was tipped to induce new flush. Tipping consisted of pruning every shoot tip on the plant, removing the tip but no leaves. One day after tipping, one branch in each tree was covered and tied with plastic bags to prevent direct contact with the foliar application. Where possible, the branch represented approximately ¼ of the canopy, selecting branches that emerged from below the lowest foliage. Soil was covered with plastic sheeting during application to prevent root uptake of OTC. OTC was applied to the entire canopy except branches shielded with plastic bags at a rate of 1 g L^-1^ of OTC (Fireline 17 WP, Agrosource, Inc., Cranford, NJ, United States), with 3.1 ml L^-1^ of Induce non-ionic surfactant (Helena Ag, Collier, TN, United States). Three hundred mL of solution per plant were applied at approximately 400 kPa, which resulted a fine mist and was sufficient to produce runoff from the leaves. One hour later, when trees were completely dried, bags were taken away. Twenty-two days after applying OTC, leaves were collected for OTC quantification. Leaves that received the direct application and those that were covered were sampled separately to assess the difference between contact and systemic delivery. Leaf samples were ground with liquid nitrogen and stored in a -20°F freezer until analysis.

### Variables Measured

Leaf gas exchange (photosynthesis, stomatal conductance, and respiration) and chlorophyll fluorescence variables were measured before heat treatment and 3 weeks after heat treatment. Leaf gas exchange was measured in young fully-mature, sun-exposed leaves. Photosynthesis was measured with in-chamber conditions at 1000 μmol PAR m^-2^ s^-1^, 400 ppm CO_2_, and 1.5 mPa vapor pressure deficit, using a portable infrared gas analyzer (Li-Cor 6800, Lincoln, NE, United States). Leaf respiration was measured in the pre-dawn dark on the same leaves. Data were recorded in the same leaf of each tree using the same system and parameters, except that the light was turned off.

For OTC quantification samples of three leaves each were collected from the portion of the canopy that received the application and from the bagged portion. Although measurable superficial residue of OTC was unlikely at 22 days after application because of photodegradation (Doi and Stoskopf, 2000), samples were rinsed for 10 s under running distilled water. Subsequently, leaves were frozen in liquid N and ground with liquid N with mortar and pestle, and approximately 600 mg samples were used for analysis. OTC was quantified by HPLC according to Hu and Wang (2016), which describes the efficiencies of OTC recovery and quantification in citrus samples. In addition to OTC concentrations, ratios of concentrations between the directly-applied and bagged samples for each tree were calculated to assess the relative degree of systemic delivery.

At the end of the experiment, leaf areas were measured. Pots were removed from the soil and roots were washed of all soil. Leaves, stems, and fine and structural roots of each tree were individually dried at 60°C for 72 h and weighed. Plant growth variables were calculated as total plant leaf area (LA), average area per leaf (mean LA), and dry weights (DW) of leaves (total leaf DW per plant), stems, fine roots, and structural roots. Total DW was calculated as the sum of all DWs, and shoot:root ratio was calculated as above-ground DWs/total root DWs.

### Data Analysis

For all variables data were analyzed for each Trial separately using the lm command in base R Stats 3.2, using a mixed model with treatments as fixed effects and block as a random effect (R Core Team, 2017). In the case of OTC concentration, subsample (direct application vs. protected leaves) was included as a fixed effect nested within plant (split-plot). For significant effects (*P* < 0.05), means separations were performed using Bonferroni’s protected least significant differences, using the agricolae package in R (Mendiburu, 2015).

## Results

### Gas Exchange

Heat treatments induced a noticeable defoliation, as is presented in the plant growth section, and one tree in the Heat-Mature Leaves treatment died in each trial. However, by the time gas exchange and chlorophyll fluorescence were measured 3 weeks later, there were no significant effects on chlorophyll fluorescence, photosynthesis, respiration, or stomatal conductance of the leaves that remained (data not shown due to non-significance of effects).

### Plant Growth

**FIGURE 2:**
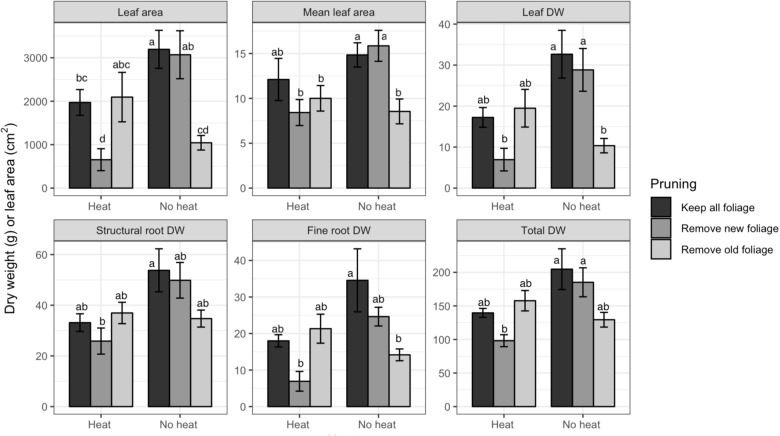
Dry weights (DW) and leaf area of various organs of Valencia/X-639 citrus plants upon termination of Trial 1, 81 days after heat treatment. Shaded bars represent means, and error bars represent standard error (*n* = 6). Bars labeled with different letters indicate significant differences at *P* < 0.05 using Bonferroni’s protected LSD.

In Trial 1 there were interactions of pruning and heat treatment for LA, mean LA, leaf DW, structural root DW, fine root DW, and total DW (*P*-values of all effects found Table 1). Leaf number was affected by heat only. For all variables, Heat reduced DW or LA accumulation (Figure 2, only variables with significant effects are shown). Within the Heat plants Mature Leaves negatively affected growth, while in No Heat plants, Young Leaves negatively affected DW or LA. Fine roots were more affected than structural roots, but the trends among treatments were the same for both variables.

**Table 1:** *P*-values of plant dry-weight variables at the end of each of two trials of heat treatment and defoliation.

Trial	Variable	Effect		
		Pruning	Heat	Pruning × heat
1	Leaf number	0.213	**0.013**	0.083
	Leaf area	0.052	**0.015**	**0.001**
	Mean leaf area	**0.036**	0.073	0.053
	Leaf dry weight	0.054	**0.008**	**0.002**
	Stem dry weight	0.549	0.126	0.058
	Structural root dry weight	0.384	**0.004**	0.054
	Fine root dry weight	**0.046**	**0.015**	**0.010**
	Total dry weight	0.169	**0.007**	**0.006**
	Shoot:root ratio	0.321	0.062	0.290
2	Leaf number	0.309	**0.048**	0.319
	Leaf area	0.571	0.198	0.940
	Mean leaf area	0.739	0.911	0.415
	Leaf dry weight	0.426	0.132	0.943
	Stem dry weight	0.869	**0.008**	0.576
	Structural root dry weight	0.569	**0.001**	0.337
	Fine root dry weight	0.707	**0.033**	0.567
	Total dry weight	0.633	**0.003**	0.822
	Shoot:root ratio	0.633	**0.003**	0.822


*Each trial consisted of *n* = 6 for all combinations and was arranged as a randomized complete block. Values less than *P* = 0.05 are in bold for clarity*.

**FIGURE 3:**
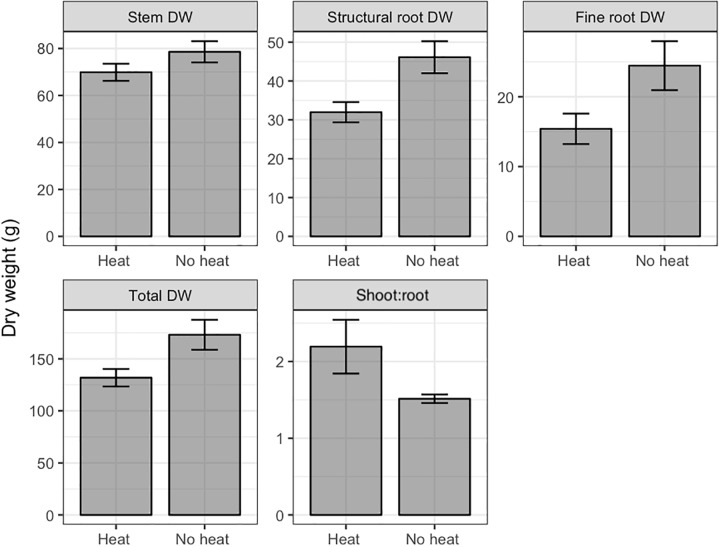
Dry weights (DW) and leaf area of various organs of Valencia/X-639 citrus plants upon termination of Trial 2, 111 days after heat treatment. Shaded bars represent means, and error bars represent standard error (*n* = 6). All contrasts significant at *P* < 0.05 using Bonferroni’s protected LSD.

In Trial 2, leaf number, stem DW, structural root DW, fine root DW, total DW, and shoot:root ratio were affected by heat treatment, while no interactions of pruning and heat treatment were found (Table 1). In all cases Heat reduced DW relative to No Heat, and increased shoot:root, by affecting roots to a greater degree than shoots (Figure 3, only variables with significant effects are shown).

### Oxytetracycline Content

There was no interaction or main effect of heat or pruning on foliar OTC content (see Table 2 for analyses of variance). However, OTC was detected in nearly all samples of leaves that did not receive direct application, while the effect of covered versus exposed leaves was significant (*P* < 0.001 in both trials; Table 3). The proportion of OTC content in covered leaves relative to the content in treatment-exposed leaves varied between the two trials. Although a direct statistical comparison between the two trials is not valid, we note that the 95% confidence intervals did not overlap.

**Table 2:** Analysis of variance of foliar oxy-tetracycline content in Valencia/X-639 after foliar application at 1 g ai. per L sampled 22 days after application.

Trial	Effect	Degrees of freedom	*F*-value	*P*-value
1	Pruning^z^	2	0.55	0.54
	Heat^y^	1	1.75	0.14
	Subsample^x^	1	24.55	< 0.0001
	Pruning × heat	2	0.34	0.71
	Pruning × subsample	2	0.44	0.65
	Heat × subsample	2	0.01	0.94
	Heat × pruning × subsample	4	0.56	0.58
2	Pruning	2	1.24	0.31
	Heat	1	2.72	0.11
	Subsample	1	295.59	<0.0001
	Pruning × heat	2	0.66	0.53
	Pruning × subsample	4	0.92	0.42
	Heat × subsample	2	0.28	0.60
	Heat × pruning × subsample	4	0.19	0.83


*^z^Pruning consisted of removal of all new leaves, removal of all mature leaves or no leaf removal. ^*y*^Heat consisted of heating above 43°C for greater than 45 s. ^*x*^Subsample contrasts leaves that received direct application with those that were shielded during application.*

**Table 3:** Oxytetracycline (OTC) concentration in leaves which received direct application or were covered during application.

Trial	Sample	OTC content (μg OTC g^-1^ FW; Mean ± SE)	Relative concentration covered leaves/uncovered leaves (95% CI)
1	Applied	0.53 ± 0.06 a^z^	
	Covered	0.21 ± 0.03 b	0.36–0.63
2	Applied	0.46 ± 0.07 a	
	Covered	0.14 ± 0.03 b	0.27–0.34


*Samples were collected 22 days after application. ^*z*^Means (*n* = 36) followed by different letters are significantly different within each trial using Bonferroni’s protected LSD (*P* < 0.05)*.

## Discussion

### Effects on Growth

Heat treatment negatively affected growth of all plant organs. In Trial 1 the interaction of heat with pruning for most growth variables may be indicative of the defoliation effect, in which many of the mature leaves senesced. This defoliation reduced the total photosynthetic capacity of the tree by reducing photosynthetic area, similar to that found in defoliation treatments by Eissenstat and Duncan (1992). This is reflected in the fact that the Heat-Young Leaves treatment had the same leaf area as Heat-All Leaves, whereas, No Heat-Young Leaves had only about 30% of the total leaf area of the other No Heat pruning treatments (Figure 2). Because LA is the primary indicator of photosynthetic capacity, all organ DWs had the same trend as LA (Figure 2). This co-occurrence of reduced DW with reduced LA did not carry over to the Trial 2 because treatments did not affect total leaf area. This may be because the trees in Trial 2 had lesser leaf area, and during a low temperature period many leaves senesced in all treatments. An important characteristic of the Heat-induced decrease in DW is that root DW decreased to a greater degree than the other DW variables (Figure 3). The prevalent conditions of primarily HLB-infected trees in Florida make root health a priority for production, because HLB has been found to dramatically reduce fine roots and thereby to limit the trees’ capacity for water uptake (Kadyampakeni et al., 2014).

### Implications for Foliar Delivery Past the Cuticle

Citrus vegetative growth occurs in spurts called flushes. In Florida, there are usually three vegetative flushes per year. Many growers consider the ideal time of application to be when flush is young (3–4 weeks old; J. Duggar, Peace River Packing, Inc., *personal communication*), based on the observation that the citrus leaf continues to deposit cuticular waxes over its entire lifespan (Syvertsen, 1982). The present results do not support that conclusion for OTC.

Reinforcing this view, studies of foliar delivery of other solutions have been more complex than a linear age-related decrease in delivery. Orbović et al. (2007) found that an adjuvant that reduced surface tension increased Cu delivery into both abaxial leaf surfaces and fruits of grapefruit (*Citrus* ×*paradisi*) as compared with adaxial surfaces, indicating that the most likely path of delivery is through stomata. A previous study by the same group found that urea penetration of grapefruit cuticles decreased between 3–4 and 6–7 week leaf age ranges, but that it increased as leaves aged beyond the 6–7 week range (Orbović et al., 2001). In that study, although cuticular wax deposition continued, much of the cuticle became disorganized, allowing many potential paths of entry of water solutions, indicating that as leaves age, non-stomatal paths of delivery increase.

This conclusion is supported by water permeance studies. Baker et al. (1975) found that citrus leaf cuticular waxes varied greatly in composition, but Geyer and Schönherr (1990) using sour orange (*Citrus aurantium*) found that a wide range of environmental conditions affected composition but did not affect permeance. They concluded that cuticular structure had a greater effect on permeance than thickness or composition within the range of citrus cuticles. Bondada et al. (2006) similarly concluded that cuticular thickness did not govern urea penetration of the cuticle of grapefruit, because, although older leaves on the same stem had thicker cuticles, urea penetration was greater in nodes 1 and 4 than 2 and 3, respectively in basipetal order. Additionally, micrographs of Orbović et al. (2001) indicate stomatal occlusion but also cuticular disruption, possibly diminishing the role of stomata and increasing the role of cuticular organization as leaves age. Thus, the present study is consistent with the body of literature, that mature and young leaves may be equally permeable to foliar-applied products, and we now add that heat treatment is unlikely to alter subsequent delivery.

### Implications for Systemic Delivery

Although few studies have addressed systemic antibiotic delivery in citrus directly, a number of studies have addressed the use of anti-microbial compounds against Las. Yang et al. (2016) compared grafting, root drench, and bark paint (at different concentrations for each method) application methods of applying several anti-microbial compounds, and concluded that bark paint at high concentrations (5–6x of commercial label concentrations) was more effective than root drench at lower concentrations. They also found that bark paints of ampicillin and actidione + validoxylamine A significantly reduced Las titer. A subsequent study found that adjuvants that improved ampicillin entry into leaves also significantly reduced Las titer under greenhouse conditions (Yang et al., 2015). Although these studies addressed delivery methods, none considered whether systemic translocation within the plant was achieved after the compound was delivered into the plant. Hu and Wang (2016) addressed translocation dynamics of trunk-injected OTC. They found that trunk injection resulted in OTC detection in leaves in all quadrants of canopy and in roots by 1-week after injection, even with a single port. These treatments greatly reduced Las titer in foliage by 4 days after injection and in roots by 14 days post-injection. A subsequent study indicated that injection of streptomycin also reduced Las titer, but to a lesser degree than OTC, though the effect of combining the two increased efficacy but was not strictly additive (Hu et al., 2017). The limitation of this study, as of others, is that it does not assess phloem-specific delivery of OTC. Phloem delivery is the objective of antibiotic application in HLB management because Las is phloem limited.

### Foliar Delivery for Control of Las

Hu and Wang (2016) applied 2 g of OTC per tree, equivalent to 2.6 foliar applications at the labeled rate of 115 g ai. ac^-1^ with 150 trees per acre. In that study, foliar OTC concentrations reached 0.6–1.5 μg g^-1^ with similar concentrations in roots, as compared to 0.14–0.21 μg g^-1^ in the present study in leaves that did not receive direct application (Table 2). In that study there was a reduction of Las titer by 4 days in foliage and by 14 days in roots, indicating that this range of concentrations was sufficient to reduce Las populations. The subsequent study compared injection rates of 1.25 and 2.5 g OTC per plant (Hu et al., 2017), with 60 and 110% reductions in Las titer respectively. Given that the 1.25 to 2.5 g injection per plant range appears to affect efficacy against Las, the lower equivalent rate of foliar application may have a much more muted effect on bacterial titers, even if a high rate of delivery into the leaf is assumed. For purposes of comparison, the recommended rate for foliar application is 1.5 lbs (681 g) per acre of product (AgroSource, Inc., Cranford, NJ, United States), or 125 g OTC per acre. At a standard planting density of 200 trees per acre would result in 0.62 g per tree in the foliar application, with lower quantities reaching the leaf apoplast, and still lower quantities entering systemic distribution. The low concentrations of OTC in tissues from the present study appear to be sufficiently below those of the trunk injection studies that they may not represent effective levels of Las control.

## Conclusion

Heat treatment had negative effects on citrus growth, and did not affect OTC uptake. Leaf age within the grouped ranges assessed in this study also did not affect systemic OTC uptake. However, OTC was delivered into the leaf and was translocated systemically. Future research should address whether tissue concentrations achieved by foliar application are sufficient to reduce Las populations, and whether the pattern of is the same between OTC and streptomycin as the two compounds registered for management of Las in citrus.

## Author Contributions

CV and NW conceived the study. MP and CV implemented the study and assessed growth and physiological variables. JL assessed the OTC content. CV, MP, and JL wrote the manuscript. NW revised the manuscript.

## Conflict of Interest Statement

The authors declare that the research was conducted in the absence of any commercial or financial relationships that could be construed as a potential conflict of interest.
